# Cell Phenotype Classification Based on Joint of Texture Information and Multilayer Feature Extraction in DenseNet

**DOI:** 10.1155/2022/6895833

**Published:** 2022-11-28

**Authors:** Shervan Fekri-Ershad, Mustafa Jawad Al-Imari, Mohammed Hayder Hamad, Marwa Fadhil Alsaffar, Fuad Ghazi Hassan, Mazin Eidan Hadi, Karrar Salih Mahdi

**Affiliations:** ^1^Faculty of Computer Engineering, Najafabad Branch, Islamic Azad University, Najafabad, Iran; ^2^Big Data Research Center, Najafabad Branch, Islamic Azad University, Najafabad, Iran; ^3^Department of Medical Laboratory Techniques, Al-Mustaqbal University College, Hillah 51001, Babylon, Iraq

## Abstract

Cell phenotype classification is a critical task in many medical applications, such as protein localization, gene effect identification, and cancer diagnosis in some types. Fluorescence imaging is the most efficient tool to analyze the biological characteristics of cells. So cell phenotype classification in fluorescence microscopy images has received increased attention from scientists in the last decade. The visible structures of cells are usually different in terms of shape, texture, relationship between intensities, etc. In this scope, most of the presented approaches use one type or joint of low-level and high-level features. In this paper, a new approach is proposed based on a combination of low-level and high-level features. An improved version of local quinary patterns is used to extract low-level texture features. Also, an innovative multilayer deep feature extraction method is performed to extract high-level features from DenseNet. In this respect, an output feature map of dense blocks is entered in a separate way to pooling and flatten layers, and finally, feature vectors are concatenated. The performance of the proposed approach is evaluated on the benchmark dataset 2D-HeLa in terms of accuracy. Also, the proposed approach is compared with state-of-the-art methods in terms of classification accuracy. Comparison of results demonstrates higher performance of the proposed approach in comparison with some efficient methods.

## 1. Introduction

Detection of cell phenotypes plays an important role in many different medical or cell biology applications. Among its most important uses, the following can be mentioned [1]:

- Analysis of the effects of genes and drugs on screening experiments
- Subcellular protein localization
- Cancer diagnosis in images acquired using cytological or histological techniques

In most related healthcare-systems and medical diagnosis processes, if the type of cells is detected automatically with high accuracy, it can increase the final performance of the related system [1]. In the last decade, fluorescence imaging is the most efficient tool to analyze the biological characteristics of cells, which is widely used by specialists [2]. Hence, cell phenotype classification in fluorescence microscopy images has received increased attention from scientists in the field of computer vision and artificial intelligence. Cells are known to exhibit complex phenotypes such as differences in shape, texture, and other qualities. The combination of intricate phenotype differences together has made cell classification and decoding biological processes a complex task. Today, in many laboratories, this task is performed by experienced and specialized persons. Therefore, it is a time-consuming process, and the detection's accuracy is not constant in different environmental and time conditions. So a cell classification task can be defined as predicting the type of input cells based on predefined cell types in the train set. The visible structures of cells are usually different from each other in terms of shape, texture, relationship between intensities, etc. Therefore, until now, various approaches have been presented for cell phenotype classification based on machine learning and computer vision techniques. In this scope, most of the efficient approaches are categorized into two main strategies [1, 3]:

- Handcrafted-based features in joint of machine learning techniques
- Deep learning-based methods

Cell phenotype classification can be categorized as visual pattern classification problems. Nearly, in all of the proposed approaches until now, feature extraction and classification have been two main phases. Feature extraction is performed before classification in machine learning-based methods. However, features are extracted during the learning process in deep-based methods. Also, in some cases, preprocessing is performed to increase final performance as a separate phase. In this respect, some approaches use low-level features, such as handcrafted, texture, color, or shape features [4]. Deep neural networks usually extract high-level features during the learning process. So, in some studies, a cell phenotype is classified based on deep learning models [5].

In the last decade, deep neural networks have grown tremendously and have provided good performance in many computer vision applications. Xu and Qiu [6] have proposed a CNN-based deep neural network for human activity recognition. They used the gradient descent algorithm to optimize deep network parameters [6]. Zheng et al. [7] proposed an innovative image denoising method based on the hybrid convolutional neural network with acronym (HDCNN). HDCNN consists of different layers, such as the dilated block, Rep VGG block, feature refinement block, and convolution layer [7]. Deep learning-based approaches are widely used in medical image analysis. Shabbir et al. [8] studied many recent studies on glaucoma detection using retinal fundus images. In [8], classical methods based on handcrafted features and deep learning-based methods are studied in terms of methodology and performance. Masood et al. [9] proposed a combination of feature selection filters in joint of different supervised classifiers for medical data analysis such as hepatitis diagnosis. Yamamoto and Miyata [10] developed a CNN for an automatic cell morphology discrimination technique based on AlexNet. A combination of AlexNet and the transfer learning technique is used in [11]. The proposed deep neural network comprised three convolution layers, two pooling layers, and two layers for providing the total combination [10]. Deep neural networks are widely used for different computer vision applications such as defect detection [12]. Latif et al. [13] presented a comprehensive review on efficient handcrafted features and deep learning approaches.

**Figure 1:**
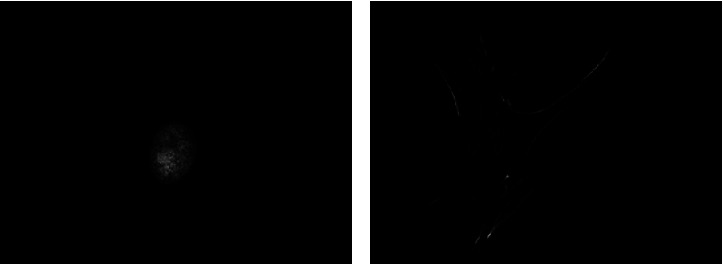
Two examples from two different classes that are completely different in terms of texture. (a) Nucleus. (b) Actin filaments.

**Figure 2:**
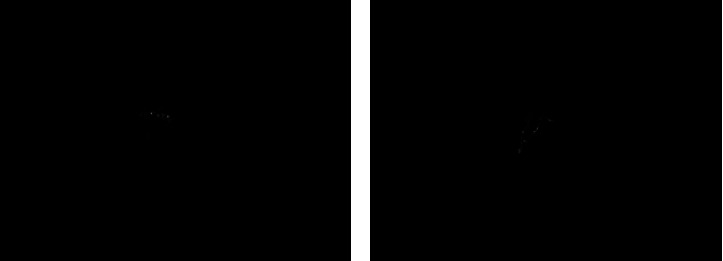
Samples of two different classes with nearly the same texture. (a) Golgi_gia. (b) Golgi_gpp.

Classification accuracy is the main performance evaluation metric in the cell phenotype classification problem. Therefore, most studies try to increase classification accuracy as a final aim. Low-level features usually extract the appearance information of the object from the image. Because the appearance of cells is different from each other, it is necessary to extract low-level features [4]. For example, two samples of two cell classes (nucleus and actin filaments) are shown in Figure 1. As can be seen, the texture of two samples is fully different. Some types of cells are not very different in terms of visual appearance, but they have differences in terms of the relationship between the intensities and texture or shape. In these cases, high-level features can play a vital role in improving final performance [5]. For example, the texture and structure of samples shown in Figure 2 are nearly the same.

In this paper, combinations of low-level and high-level features are used in the learning process. First of all, the operator-improved local quinary pattern (ILQP) [14] is used to extract texture features. Next, a multilayer method is presented to extract high-level and deep features using DenseNet [15]. Finally, the normalized combinations of these features are used to feed the classifier. The main novelty of this paper is to use the combination of texture and deep features to classify the types of cells. An improved local quinary pattern (ILQP) is one of the most efficient operators for image texture description, which was developed in 2020. In this article, for the first time, ILQP has been used to extract texture features of cells. Also, in this paper, the classification layers in DenseNet are removed and replaced by the flatten layer. The study of published articles shows that for the first time, the combination of deep features extracted from DenseNet in joint of ILQP features has been used for medical image analysis.

In this respect, the main contribution of this paper can be mentioned as follows:

- Cell phenotype classification based on the combination of low-level texture features and high-level features extracted using DenseNet provides higher performance in comparison with most exciting approaches that use one of the feature types
- Extracting deep features from the dense blocks of DenseNet in a multilayer format provide discriminative features for cell phenotype classification

## 2. Related Works

As mentioned above, the problem of cell phenotype classification is grouped as pattern classification problems. Therefore, most of the methods that have been presented so far can be categorized into two groups, methods based on machine learning and methods based on deep learning [1]. In most of the machine learning-based methods, feature extraction and classification phases are separate from each other, which is due to the nature of these types of classifiers. In the methods presented based on deep learning, the feature extraction process is performed in the learning phase, and the classification process is performed in the last layer of the deep network. In the following section, some effective methods in these two fields are examined.

In a study, Nanni et al. [16] proposed a three-step method for cell image classification. In the first step, rough segmentation is performed to crop background regions. The authors in [16] suggested the first step to improve final performance. Next, feature extraction is performed in the second step. They used basic LBP as a texture analysis operator in this step. The histogram of LBP values in different color channels of the segmented image is quantized to extract numerical feature vectors. In the experiments, Haralick features and TAS feature sets are also performed in joint of LBP. Finally, the classification phase is performed in the third step. They suggested using the SVM and random subspace neural network (RSNN) as classifiers for their experiments.

Liu et al. [17] proposed an approach for cell classification in microscopic images based on handcrafted texture features. In this respect, a novel LBP-like operator is presented by assigning an adaptive neighborhood radius in the primary LBP process. Next, a spatial adjacent histogram technique is used to encode microstructures. Some evaluations are performed by the authors in [17] on different medical datasets such as the HeLa cell phenotype dataset. The reported results show higher performance for the proposed operator than for the primary LBP version and some other texture-based operators.

Fekri-Ershad [18] proposed a new LBP-like operator called multithreshold uniform local ternary patterns (MT-ULTPs) to extract discriminative texture features. As reported in [18], the noise sensitivity of MT-ULTP is lower than that of many other LBP-like operators, such as LTP [19], because of using different binarization thresholds in the local pattern building process. Also, it can be performed in different radius sizes to extract micro- and macro-local patterns. MT-ULTP in joint of the random forest as a classifier provides an 86.77 percent accuracy for cell phenotype classification for the 2D-HeLa dataset [18].

An ensemble method of different CNNs in terms of depth and structure is used in combination with a feature concatenation technique [20] for medical image classification. Also, a PCA technique (with 95 percent) and transfer learning are performed to improve the final performance of the proposed ensemble method. The authors evaluated the performance of their proposed approach on the Pap smear and cell phenotype images. Results show the classification accuracy about 93.51 percent for the 2D-HeLa cell dataset [20]. The standard deviation of the classification accuracy was 2.29 percent, which is not suitable for medical diagnosis problems.

Zhang and Zhao [21] used the CapsNet neural network for classification of ten phenotypes of cells. They used 3 capsules in the structure of CapsNet which performed convolution and reshape and lambda layers in a sequence format. Capsules in CapsNet were trained to capture the possibility of certain features and variants rather than to capture the characteristics of a specific variant. The redesigned CapsNet in [21] provided a classification accuracy of 93.08 for the 2D-HeLa cell phenotype dataset.

Nguyen et al. [22] presented an innovative deep network architecture using transfer learning for microscopic image classification such as cells. They concatenated total features that are extracted using 3 different pretrained CNNs in the learning phase. Finally, two fully connected layers are fed using these features to perform the classification phase. Inception-V3, ResNet152, and Inception-ResNet-V2, are three pretrained CNNs that are used in the learning phase [22]. High complexity and respected higher runtime, because of using three CNNs, are two main limitations of the proposed method [22]. Also, the number of parameters of this method is too high. So pretrained weights may not provide high accuracy in other related applications.

## 3. Proposed Cell Phenotype Classification Approach

**Figure 3:**
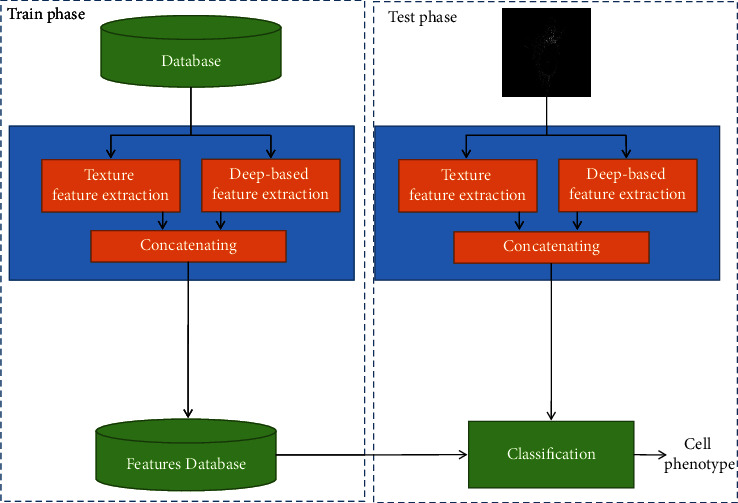
The main block diagram of the proposed approach.

As mentioned above, cell phenotype classification can be categorized as visual pattern classification. Therefore, our proposed approach included two main phases, feature extraction and classification. The block diagram of our proposed approach is shown in Figure 3. Each box is described with details in the following sections.

### 3.1. Improved Local Quinary Patterns

Texture is one of the main properties which humans use to define images. The image texture provides efficient features for content, objects, background, contrast, internal relationship, etc [23]. As shown in Figure 1, different types of cells differ from each other in terms of their visual texture. Therefore, extracting texture features may play an important role in cell classification. Until now, various methods have been presented for image texture analysis. Most of the proposed operators so far have described the image texture as a set of numerical features. The method presented in this paper does not depend on a specific type of texture analysis operator. Therefore, most of the operators that can extract numerical features can be performed.

The local binary pattern (LBP) is one of the most efficient descriptors to extract texture features. The initial version of this operator was presented in 2000 by Ojala et al. [24]. In the last two decades, many different modified versions of this operator have been presented, each trying to cover each other's weaknesses. One of the most recent LBP-like operators that provided acceptable performance in texture analysis is an improved local quinary pattern (ILQP) [14]. The initial version of LBP produces binary patterns in each local neighborhood. Nanni et al. [25] suggested a quinary pattern to reduce the noise sensitivity of the initial LBP version called the local quinary pattern (LQP). An initial LBP operator uses the central point intensity as a fixed threshold to produce binary patterns:

(1) $$\begin{array}{c} {LBP}_{P,R}=\overset{P-1}{\underset{i=0}{\sum }}\rho \left(g_{i}-g_{c}\right)2^{i}, \end{array}$$

(2) $$\begin{array}{c} \rho \left(x\right)=\left\{\begin{array}{cc} 1\text{,} & g_{i}-g_{c}>0\text{,}\\ 0\text{,} & \text{otherwise.} \end{array}\right. \end{array}$$

Therefore, even weak noise, if it is placed on the image, leads to a change in the intensity of pixels (including neighborhood centers), and the efficiency of the operator drops drastically. LQP uses two fixed thresholds (*T*_1_ and *T*_2_) to create a quinary pattern with 5 digits [25]. It is performed using a new transform function as follows:

(3) $$\begin{array}{c} \rho \left(g_{c},g_{i},T_{1},T_{2}\right)=\left\{\begin{array}{cc} \begin{array}{c} 2,\\ 1,\\ \begin{array}{c} 0,\\ \begin{array}{c} -1,\\ -2, \end{array} \end{array} \end{array} & \begin{array}{c} g_{i}\geq g_{c}+T_{2},\\ g_{c}+T_{1}\leq g_{i}<g_{c}+T_{2},\\ \begin{array}{c} g_{c}-T_{1}\leq g_{i}<g_{c}+T_{1},\\ \begin{array}{c} g_{c}-T_{2}\leq g_{i}<g_{c}-T_{1},\\ \text{otherwise.} \end{array} \end{array} \end{array} \end{array}\right. \end{array}$$

In order to reduce complexity, Nanni et al. [25] proposed a transform function *B*_c_(x) to convert LQP into four binary patterns with acronym *B*_2_, *B*_1_, *B*_−2_, and *B*_−1_:

(4) $$\begin{array}{c} B_{c}\left(x\right)=\left\{\begin{array}{cc} 1\text{,} & x=c\text{,}\\ 0\text{,} & \text{otherwise.} \end{array}\right.c\in \left\{2,1,-1,-2\right\}\text{.} \end{array}$$

**Figure 4:**
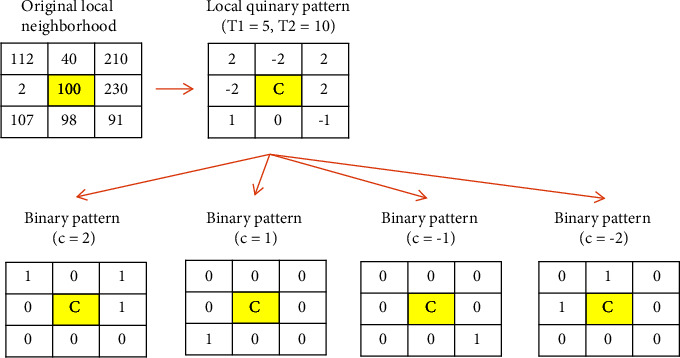
An example of the LQP process and converting the quinary pattern to four binary patterns.

Similar to LBP, each binary pattern is mapped to decimal numbers. Therefore, four decimal numbers can be produced for each neighborhood. Finally, four histograms can be computed for each image, which can be used as a texture feature set. An example of converting the quinary pattern to four binary patterns is shown in Figure 4.

An improved local quinary pattern (ILQP) is proposed in [14] to reduce limitations of LQP operators. As mentioned above, thresholds should be selected by users in the LQP process. A dynamic algorithm is described to define LQP's thresholds in ILQP [14]. To choose the first threshold (*T*_1_), the global median absolute deviation (GMAD) is suggested for ILQP. GMAD is a statistical criterion that measures how a set of data (such as intensities) is developed. GMAD is calculated as the average of local MADs over the whole image, so it is not sensitive to noise. If noise causes changes in intensities of some pixels, the effect of disturbance on the average of the total data (all pixels) will be very low. GMAD can be calculated as follows:

(5) $$\begin{array}{c} GMAD=median\left(\left|lmad-median\left(lmad\right)\right|\right)\text{,} \end{array}$$

(6) $$\begin{array}{c} lmad=\left\{{LocalMAD}_{k}|k=1,2,\ldots ,N\right\}\text{,} \end{array}$$

(7) $$\begin{array}{c} \text{LocalMAD}=median\left(\left|G-median\left(G\right)\right|\right),where:G=\left\{g_{i}|i=0,1,\ldots ,P-1\right\}\text{,} \end{array}$$

where *P* is the total number of neighbors in each local neighborhood and *g*_*i*_ shows the intensity value of the *i*^th^ neighbor. Also, *N* is the total number of possible neighborhoods in the image based on the radius of the ILQP operator. The global significant value (GSV) was proposed for the first time by the authors in [14] for medical image analysis. However, in some other studies, it is used as a discriminative statistical feature in learning phases. It is suggested that GSV should be considered as the second threshold (*T*_2_) in the ILQP process. GSV can be computed based on the local significant value (LSV). LSV calculates the difference between intensity of neighboring centers and surrounding neighbors. The total average of LSV for the whole images is called GSV. It can be calculated as follows:

(8) $$\begin{array}{c} {LSV}_{c}=\frac{1}{P}\overset{P-1}{\underset{i=0}{\sum }}\left(\left|g_{c}-g_{i}\right|\right)\text{,} \end{array}$$

(9) $$\begin{array}{c} GSV=\frac{1}{M\times N}\overset{N}{\underset{i=1}{\sum }}\overset{M}{\underset{j=1}{\sum }}{LSV}_{i,j}, \end{array}$$

where *M* × *N* shows the image size. Also, LSV_*i*, *j*_ means the local significant value of the neighborhood with a center pixel *c* with coordination (*i*, *j*).

### 3.2. Proposed Multilayer Feature Extraction in DenseNet

DenseNet was proposed in the CVPR conference (2017) as an innovative CNN-based structure [26]. DenseNet, instead of most CNN-based networks such as ResNet, CIFAR, ImageNet, and AlexNet, utilizes dense connections between internal layers using dense blocks. DenseNet contains shorter connections between its layers close to the input and those close to the output. Therefore, in most cases, DenseNet can be more accurate in the training process. ResNet [27] uses skip connections that bypass the nonlinear transformation, but DenseNet adds a direct connection from any layer to any subsequent layer. So the *i*^th^ internal layer receives the feature maps of all former layers. Therefore, all layers can be connected (with matching feature map sizes) directly to other ones.

In recent works, DenseNet has provided acceptable results for classification problems such as medical diagnosis. The main reason for the success of this network is that it is deeper than some common networks and its optimization is easier. DenseNets have some advantages such as

- Alleviate the vanishing-gradient problem
- Strengthen feature propagation
- Encourage feature reuse
- Lower parameters in comparison with existing networks

The main novelty of the DenseNet architecture is to collect dense blocks. Each version of DenseNet consists of 4 dense blocks with varying number of layers. In this respect, different versions of DenseNet are developed. Structures of some efficient DenseNet versions are shown with details in Table 1. As can be seen, the main difference is in the number of internal layers of each dense block. Also, feature maps are highlighted in Table 1 in terms of output size.

**Figure 5:**
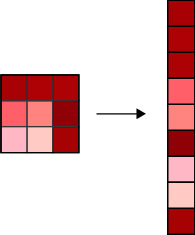
A visual example of the flattening layer process.

The basic DenseNet framework contains four dense blocks, with each block comprising four convolution layers. Furthermore, each convolution layer applied four techniques batch normalization (BN), ReLU activation, squeeze, and excite operations. According to the initial filter size and stride value, after each step, the feature vector size increases due to the concatenation operation. The transition layer was introduced after each DB to perform the downsampling step to solve this problem. The *N*^th^ layer of the framework has *N* inputs as the *N*^th^ layer takes the outputs of all previous *N* − 1 layers:

(10) $$\begin{array}{c} I_{N}=H_{N}\left(\left[I_{0},I_{1},I_{2},I_{N-1}\right]\right)\text{,} \end{array}$$

where [*I*_0_, *I*_1_, *I*_2_,…, *I*_*N* − 1_] are the feature maps from the previous *N* − 1 layers, which are connected to the *N*^th^ layer and indicated by *I*_*N*_. Furthermore, the transition layer comprised convolution and pool layers. The bottleneck layer contained a 1 × 1 convolution layer, which was employed to minimize the size of feature maps and enhance computational efficiency. In most cases, the final feature map of DenseNet is used to classify the input image. In this study, we suggested using multilayer feature maps in a concatenating format for the classification phase. The output feature map of each dense block is first entered into a new average pooling layer, and after dimension reduction, the output is entered into a flatten layer. The flattening process is commonly used to convert all the resultant two-dimensional feature maps from pooling/convolutional layers into a single long continuous linear vector. The flattened matrix is fed as input to the fully connected layer to classify the images in CNNs. As a result, the feature map in the matrix format is converted to a feature vector in low dimensions. A visual example of the flatten layer is shown in Figure 5.

**Figure 6:**
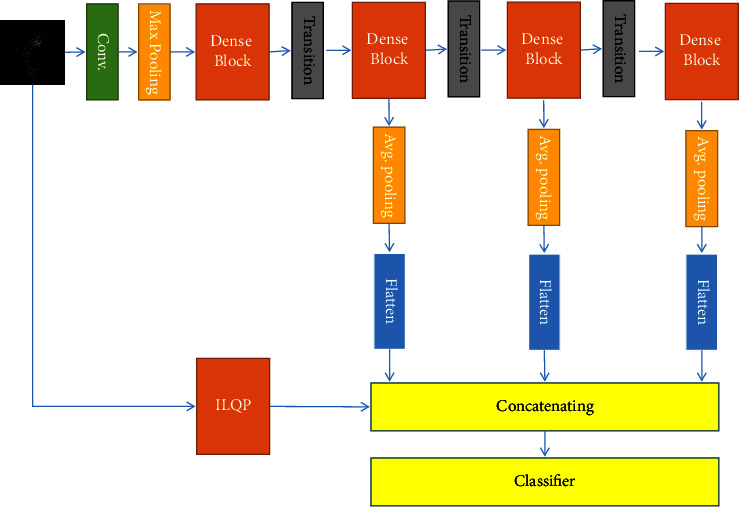
The block diagram of the proposed approach.

The primary layers in all CNN-based networks usually show low-level and low-depth properties of the image. In our proposed approach, low-level features are extracted using the ILQP operator. Therefore, to reduce the dimensionality and computational complexity, the output of the first dense block is not included in the suggested feature extraction process. The structure of our proposed feature extraction approach is shown in Figure 6. The main innovation of this paper is to combine texture features and deep high-level features to classify cell types. Figure 3 shows the train and test phase of the proposed method with focus on innovation. As described in this section, to extract deep high-level features, the structure of the classification layers in DenseNet has been removed and new layers have been added. In Figure 6, the feature extraction phase and how to feed the classifier are shown with focus on this point. As described, classification layers of DenseNet have been removed in the proposed approach. Therefore, we did not use them directly for classification. In other words, DenseNet is used to extract deep high-level features in our proposed approach. Feature maps are not very different in DenseNet versions that do not differ much in terms of the number of layers. Therefore, four versions of DenseNet (D.Net-121, D.Net-169, D.Net-201, and D.Net-264) have been selected in such a way that the number of internal layers that play a role in making the feature map increases from low to high.

As described above, four different DenseNet versions are evaluated in this paper, which are DenseNet-121, DenseNet-169, DenseNet-201, and DenseNet-264. All the above networks have been trained by Adam optimizer in 20 epochs. The focal loss function with *γ* = 2 is used for the training process [28, 29]. The initial learning rate is considered to be 10^−5^ for first 10 epochs. Then, it decreases to 10^−6^ for next 10 epochs. We select the best loss function, which is a hyperparameter, which depends on the problem we are facing. Multiclass cross-entropy loss seems a wise choice for many studies. However, according to the amount of distribution of cells in the body of each human and according to the database samples, we are facing an imbalanced classification problem. Focal loss is another choice that we can leverage its properties to enhance the performance of our model. This loss function tries to generate the class-weighting system in order to balance the samples in each batch size of data (Equation (11)):

(11) $$\begin{array}{c} FL\left(p_{t}\right)=-\alpha_{t}{\left(1-p_{t}\right)}^{\gamma }\log\left(p_{t}\right)\text{.} \end{array}$$

Here, pt is a function of true labels. Focal loss can be interpreted as a binary cross-entropy function multiplied by a modulating factor (1 − *pt*) *γ* which reduces the contribution of easy-to-classify samples. Weighting factors *α*_*t*_ balance the modulating factor. There are several approaches for incorporating focal loss into a multiclass classifier. One-versus-the-rest (OvR) technique is used in this paper, in which a binary classifier is trained for each class *C*. The data from class *C* are treated as positive, and all other data are treated as negative.

## 4. Experimental Results

### 4.1. Dataset

**Figure 7:**
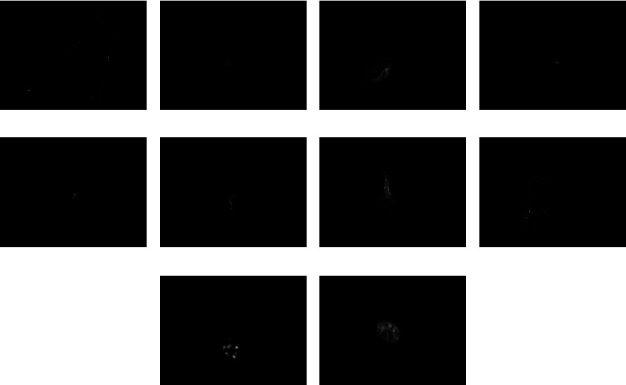
Some samples of the HeLa dataset in different 10 classes. (a) Actin filaments. (b) Endosome. (c) ER. (d) Golgi_gia. (e) Golgi_gpp. (f) Lysosome. (g) Microtubules. (h) Mitochondria. (i) Nucleolus. (j) Nucleus.

To evaluate the performance, experiments were carried out on benchmark cell phenotype datasets called 2D-HeLa [30] and human epithelial type-2 (Hep2). The 2D-Hela database contains 862 images that are categorized into 10 classes of cell types. The size of all images is the same and equal to 382 × 382. All images are saved in the TIFF format. The size of classes is not balanced, and there are between 73 and 98 samples in each class. The vertical and horizontal resolutions of all images are 72dpi and scale variation can be seen in images. Some examples of the 2D-HeLa dataset are shown in Figure 7.

**Figure 8:**
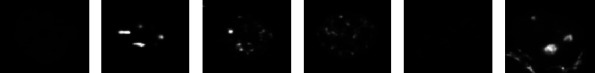
Some examples of the Hep2 dataset. (a) Homogeneous. (b) Nucleolar. (c) Centromere. (d) Speckled. (e) Nuclear membrane. (f) Golgi.

The human epithelial cell type 2 (Hep2) dataset contains 63,445 cell images in six classes. This dataset is collected by single-cell segments from 948 images from a set of multicell images submitted at the ICPR 2014 HEp-2 cell competition. The classification task in this paper is to distinguish different staining patterns in Hep2 images with indirect fluorescence to indicate antibodies associated with autoimmune diseases. The six classes in this dataset are homogeneous (HO), speckled(SP), nucleolar(Nuc), centromere(CE), Golgi(GO), and nuclear membrane(Num). Some examples of Hep2 dataset samples are shown in Figure 8.

### 4.2. Performance Evaluation Metrics

As mentioned above, cell phenotype classification can be categorized as visual pattern classification problems. Therefore, accuracy is the main metric to evaluate the performance of related studies. Cell classification is a multiclass problem. Hence, the classification accuracy can be calculated using the confusion matrix as the sum of correct cells in the table (the number of correct label-predicted test samples) divided by all cells in the table (total number of test set samples). In other words, accuracy is the most intuitive performance measure, and it is simply a ratio of correctly predicted observation to the total observations. Also, the performance of our proposed approach is evaluated in terms of precision and *F*1 score. All of the performance- evaluated measures are described in Equations (12)–(15), where TP means true positive, FP means false positive, and FN shows false negative. Cell phenotype classification is a multiclass classification problem, so measures are evaluated as an average of all classes:

(12) $$\begin{array}{c} accuracy=\frac{correctly\;predicted\;observation}{total\;observation}\times 100\text{,} \end{array}$$

(13) $$\begin{array}{c} precision=\frac{TP}{TP+FP}\text{,} \end{array}$$

(14) $$\begin{array}{c} recall=\frac{TP}{TP+FN}\text{,} \end{array}$$

(15) $$\begin{array}{c} F1-score=\frac{2\times \left(\text{precision}\times \text{recall}\right)}{\text{recall}+\text{precision}}\text{.} \end{array}$$

### 4.3. Performance Evaluation of the Proposed Approach

Multilayer feature extraction in DenseNet is proposed in this paper. Our proposed approach can be performed in each type of DenseNets. Only the number of extracted features may be different. In this respect, we evaluated our proposed approach based on different DenseNet types in terms of accuracy. The combination of texture and deep features is extracted in this paper. Therefore, most of the numerical-based classifiers can be used in the classification phase. We evaluated our proposed approach using different efficient classifiers as mentioned in Tables 2 and 3.

As can be seen in Tables 2 and 3, DenseNet169 provides the highest accuracy compared with other versions. As mentioned above, the Adam optimizer with the focal loss function is used in this paper to optimize hyperparameters. The effectiveness of the proposed method is evaluated based on different learning rates, and finally, a learning rate of 10^−5^ in the first 10 epochs and a learning rate of 10^−6^ in the next 10 epochs provided the highest classification accuracy. In Table 4, the evaluation results of the proposed method are presented based on DenseNet169 in terms of different learning rates.[31].

### 4.4. Comparison with State-of-the-Art Methods

The performance of the proposed method is compared with some efficient approaches in terms of accuracy as reported in Tables 5 and 6. In this respect, to have a fair comparison, the same validation conditions (*K*-folds) and same dataset (2D-HeLa and Hep2) are considered for comparison experiments. In order to have a fair comparison, all of the compared results in Tables 5 and 6 are reported based on results in related papers. In some studies, standard deviation is not reported. Efficient and up-to-date methods have been chosen from both common strategies (handcrafted and deep-based). The comparison results show that our proposed method provides a higher accuracy rate than other state-of-the-art methods in the related scope. The ensemble method of inception-v3, ResNet152 and inception-ResNet-v2 [20] provides about 0.15 percent higher accuracy than our proposed approach. As can be seen, the standard deviation of our proposed approach is less than about 0.44 percent [20], which shows that our proposed approach is more stable in experiments. Also, the proposed approach in [20] is an ensemble method of three different CNNs, which increase the runtime and computational complexity. In this paper, a combination of handcrafted texture features and deep features extracted from modified DenseNet has been used to classify cell types. Cell-type classification is performed on the same database using three types of pretrained DenseNet. The related results are shown in Table 5 as the baseline. The results show that the proposed combinational method provides higher accuracy than baseline DenseNet. Also, the performance of the proposed approach is evaluated in terms of precision and *F*1 score. Some of the compared methods reported their performance in terms of *F*1 score as can be seen in Tables 5 and 6.

## 5. Conclusion

The main goal of this study was to propose an efficient approach for cell phenotype classification. Unlike most popular approaches, low-level and high-level features are performed in joint of format in the learning phase. ILQP is performed to extract handcrafted texture features. In order to extract high-level features, an innovative multilayer feature extraction method based on DenseNet is proposed. The proposed approach provides about 95.59 percent classification accuracy for the Hep2 dataset and 93.36 percent accuracy for the 2D-HeLa dataset. The experimental results show the higher performance of the proposed approach compared with that of state-of-the-art methods in this literature for HeLa and Hep2 datasets. Also, the results prove that the combination of texture and deep features has higher accuracy than using the baseline deep network alone, with more than 0.7 percent for benchmark cell datasets. The runtime of the proposed approach is lower than that of popular CNN-based methods because of removing fully connected and softmax layers from the end of the deep structure. One of the important advantages of the presented method is its generalizability to all kinds of feature extraction operators. The proposed method for combining texture and deep features has a general structure. It means that instead of the ILQP operator, any other operator that extracts features in vector form can be used to combine with deep information. The method presented in this paper has two phases of learning and classification. Therefore, as a suggestion for future works, it can be used for many different problems of visual pattern classification for the computer vision scope.

## Figures and Tables

**Table 1:** The structure details of four used DenseNet versions.

Layers	Output size	DenseNet-121		DenseNet-169		DenseNet-201		DenseNet-264	
Convolution	112 × 112	7 × 7 conv., stride 2							
Pooling	56 × 56	3 × 3 max pool, stride 2							
Dense block (1)	56 × 56	1 × 1 conv 3 × 3 conv	×6	1 × 1 conv 3 × 3 conv	×6	1 × 1 conv 3 × 3 conv	×6	1 × 1 conv 3 × 3 conv	x6

Transition layer (1)	56 × 56	1 × 1 conv							
	28 × 28	2 × 2 average pool, stride 2							
Dense block (2)	28 × 28	1 × 1 conv 3 × 3 conv	×12	1 × 1 conv 3 × 3 conv	×12	1 × 1 conv 3 × 3 conv	×12	1 × 1 conv 3 × 3 conv	×12

Transition layer (2)	28 × 28	1 × 1 conv							
	14 × 14	2 × 2 average pool, stride 2							
Dense block (3)	14 × 14	1 × 1 conv 3 × 3 conv	×24	1 × 1 conv 3 × 3 conv	×32	1 × 1 conv 3 × 3 conv	×48	1 × 1 conv 3 × 3 conv	×64

Transition layer (3)	14 × 14	1 × 1 conv							
	7 × 7	2 × 2 average pool, stride 2							
Dense block (4)	7 × 7	1 × 1 conv 3 × 3 conv	×16	1 × 1 conv 3 × 3 conv	×32	1 × 1 conv 3 × 3 conv	×32	1 × 1 conv 3 × 3 conv	×48

Classification layer	1 × 1	7 × 7 global average pool							
		1000D fully connected, softmax							

**Table 2:** The performance evaluation of the proposed approach based on different classifiers and different DenseNet types in terms of accuracy (%) on the HeLa dataset.

DenseNet type	Classifier					
	1-NN	3-NN	5-NN	MLP	Random forest	SVM
121	84.25	84.94	84.73	89.28	87.54	90.18
169	85.28	86.73	86.08	93.36	92.49	92.75
201	85.17	86.06	85.97	91.85	90.92	91.18
264	84.39	85.12	84.67	89.47	88.28	90.07

**Table 3:** The performance evaluation of the proposed approach based on different classifiers and different DenseNet types in terms of accuracy (%) on the Hep-2 dataset.

DenseNet type	Classifier					
	1-NN	3-NN	5-NN	MLP	Random forest	SVM
121	85.91	86.64	86.68	91.52	90.10	91.24
169	86.90	88.90	87.88	95.59	95.06	95.36
201	86.87	87.76	87.90	94.08	93.46	93.75
264	86.11	86.42	86.37	91.33	90.74	91.30

**Table 4:** The performance evaluation based on different learning rates.

Learning rate (first 10 epochs)	Learning rate (second 10 epochs)	Weight decay value	Accuracy (%)
10^−3^	10^−4^	10^−3^	93.21
10^−3^	10^−4^	10^−2^	93.37
10^−3^	10^−5^	10^−3^	94.29
10^−3^	10^−5^	10^−2^	94.42
10^−4^	10^−5^	10^−3^	94.81
10^−4^	10^−5^	10^−2^	94.93
10^−5^	10^−6^	10^−3^	95.14
10^−5^	10^−6^	10^−2^	95.59
10^−5^	10^−5^	10^−3^	94.98
10^−5^	10^−5^	10^−2^	95.08

**Table 5:** Comparison results of state-of-the-art methods on the 2D-HeLa dataset in terms of accuracy, precision, and *F*1 score (%).

Approach	Type of features	Accuracy (%)	Precision (%)	*F*1 score (%)
SAHLBP (BoW (VQ) + SPM + SVM) [17]	Handcrafted	84.49 ± 2.2	—	83.75
SIFT + SAHLBP (BoW (VQ) + SPM + SVM) [17]	Handcrafted	86.20 ± 2.5	—	85.35
MLBP_8,1_ + MLBP_16,2_ [18]	Handcrafted	84.33	—	—
MT-ULTP_8,1_ + MT-ULTP_16,2_ [18]	Handcrafted	86.77	—	—
Haralick-based SVM [21]	Handcrafted	84.10	—	—
Random subspace of LMC [32]	Handcrafted	90.24	—	—
AdaBoost ERC [32]	Handcrafted	91.53 ± 0.02	—	—
SIFT (BoW (LLC) + SPM + softmax) [33]	Handcrafted	89.37 ± 1.5	—	—
LTP_16,2_ [34]	Handcrafted	87.00	—	—
LBP-rotation invariant uniformLBP_16,2_ [34]	Handcrafted	82.70	—	—
Orthogonal locality preserving projection (OLPP) [34]	Handcrafted	89.30	—	—
Neighborhood preserving embedding (NPE) [34]	Handcrafted	93.20	—	—
Discriminative LBP [34]	Handcrafted	84.5	—	—
LBP-rotation invariant [34]	Handcrafted	75.01	—	—
Completed LBP [34]	Handcrafted	88.8	—	—
Random subspace ensemble of Levenberg–Marquardt neural network (RSNN) [16]	Classic neural nets	85.00	—	—
VGG-16 [1]	Deep	72.10 ± 3.98	—	—
VGG-16 + transfer learning [1]	Deep	87.07 ± 2.86	—	—
Inspection-v3 [1]	Deep	83.18 ± 2.88	—	—
Ensemble inception-v3 and Resnet152 and inception Resnet-v2 [20]	Deep	93.51 ± 2.29	—	—
Ensemble inception-v3 and Resnet152 [20]	Deep	92.56 ± 1.92	—	—
CapsNet [21]	Deep	93.08	—	—
Inspection-v3 + transfer learning [22]	Deep	90.72 ± 1.85	—	—
Inspection ResNet-v2 [22]	Deep	92.00 ± 1.97	—	—
DenseNet121 (baseline)	Deep	91.87 ± 2.05	91.75	91.04
DenseNet169 (baseline)	Deep	92.63 ± 1.63	92.37	92.03
DenseNet201 (baseline)	Deep	91.72 ± 1.48	91.78	91.59
DenseNet264 (baseline)	Deep	90.98 ± 1.96	90.36	90.68
ILQP + ML-DenseNet (proposed approach)	Handcrafted + deep	93.36 ± 1.85	93.39	93.13

**Table 6:** Comparison results of state-of-the-art methods on the Hep2 dataset in terms of accuracy, precision, and *F*1 score (%).

Approach	Type of features	Accuracy (%)	Precision (%)	*F*1 score (%)
VGG-16 [1]	Deep	86.36 ± 0.84	—	85.00
VGG-16 + transfer learning [1]	Deep	86.91 ± 0.50	—	90.00
Inspection-v3 [1]	Deep	82.37 ± 0.36	—	81.00
Inspection-v3 + transfer learning [1]	Deep	84.65 ± 0.42	—	89.00
Scale space + SVM [17]	Handcrafted	87.99	—	84.05
Ensemble inception-v3 and Resnet152 and inception-Resnet-v2 [20]	Deep	94.98 ± 1.13	—	—
Ensemble inception-v3 and Resnet152 [20]	Deep	94.78 ± 1.05	—	—
ResNet152 [20]	Deep	92.28 ± 1.59	—	—
Deep autoencoder [35]	Deep	88.80	—	—
DenseNet121 (baseline)	Deep	94.22 ± 2.10	94.16	93.94
DenseNet169 (baseline)	Deep	94.85 ± 1.12	94.42	94.15
DenseNet201 (baseline)	Deep	94.16 ± 1.79	94.17	94.02
DenseNet264 (baseline)	Deep	93.27 ± 1.64	93.07	93.17
ILQP + ML-DenseNet (proposed approach)	Handcrafted + deep	95.59 ± 1.02	95.61	95.28

## Data Availability

The data used to support the findings of this study are available from the corresponding author upon request.
